# Tracheocele: A Rare Entity

**DOI:** 10.22038/IJORL.2022.53313.2815

**Published:** 2022-07

**Authors:** Souha Kallel, Mohamed Amin Chaabouni, Wadii Thabet, Malek Mnejja, Khaireddine Ben Mahfoudh, Ilhem Charfeddine

**Affiliations:** 1 *Department of ENT and Cervicofacial Surgery. Habib Bourguiba’s Teaching Hospital, El Ferdaous Avenue, 3029 Sfax, Tunisia. University of Sfax. *; 2 *Department of Radiology. Habib Bourguiba’s Teaching Hospital, El Ferdaous Avenue, 3029 Sfax, Tunisia.*

**Keywords:** Computed tomography scan, Diverticulum, Tracheocele, Tracheal diseases

## Abstract

**Introduction:**

Tracheocele or tracheal diverticulum is an uncommon benign entity that can be congenital or acquired. It is usually diagnosed incidentally on cervicothoracic imaging. Our aim is to describe the etiopathogenic, clinical and paraclinical features of the tracheocele as well as its therapeutic modalities.

**Case Report:**

We report 2 cases of asymptomatic congenital tracheocele occurred in a boy and a woman, incidentally found on cervical CT scan done for accidental ingestion of chicken bone and infected thyroid hematocele respectively. The tracheocele, in our 2 cases, was probably congenital: no risk factors were noted and the opening of the tracheocele was narrow. The tracheocele was located in the right posterolateral tracheal wall in the 2 cases. It communicated with the tracheal lumen in one case. The female patient underwent a right lobectomy and resection of the tracheocele. For the boy, our attitude was conservative. The evolution was uneventful in the 2 cases.

**Conclusions:**

The presence or absence of risk factors, CT scan, bronchoscopy and histologic exam may distinguish between congenital and acquired forms. Asymptomatic patients are managed conservatively. Surgical resection is the treatment of choice for symptomatic patients.

## Introduction

Tracheocele or tracheal diverticulum is an air sac arising from the wall of the trachea (1,2). It is a rare condition that can be congenital or acquired (1,2). The incidence of tracheoceles is 2.4% (1,3). Congenital forms are less frequent than the acquired ones. Compared to congenital tracheocele , acquired form has a thin wall and a wide opening (1,3,4). Chronic cough is the main cause of acquired tracheocele. This entity is often asymptomatic and mostly found fortuitously on thoracic or cervical imaging (1,3). The diagnosis is confirmed by computed tomography (CT) scan and/or tracheoscopy. The treatment options include: observation, fine needle aspiration, surgical resection and endoscopic treatment (2).

The purpose of this paper is to describe the etiopathogenic, clinical and paraclinical features of this condition as well as its therapeutic modalities.

## Case Reports

***Case 1***

A 14-year-old boy with no past medical history presented with odynophagia lasting for 2 days after accidental ingestion of chicken bone. He gave no history of dyspnea or dysphagia. He had not a fever or chest pain. There was no history of chronic cough or repeated respiratory tract infections. Oropharynx examination was normal. There was no cervical subcutaneous emphysema. Indirect hypopharyngoscopy was normal. Chest X-ray was normal. 

**Fig 1:**
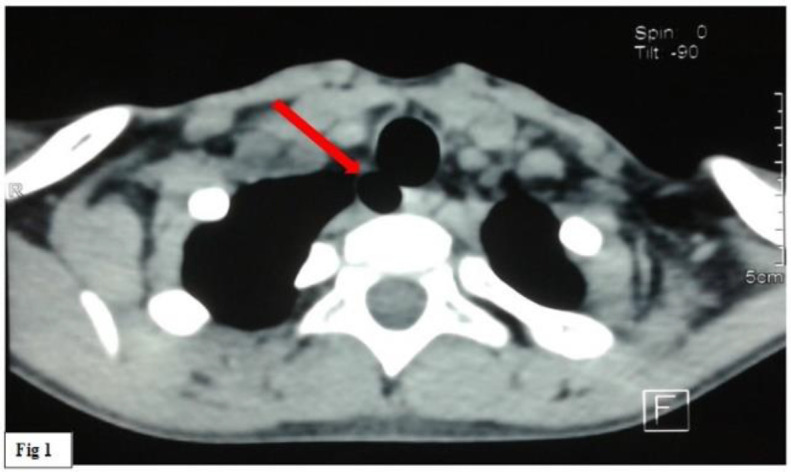
Cervical CT (computed tomography) scan in axial section shows a 11 mm air image located in the right posterolateral tracheal wall, at the thoracic inlet. It communicates with the tracheal lumen

A cervico-thoracic CT scan was performed and did not show a foreign body. However, it revealed incidentally a 11 mm air image located in the right posterolateral tracheal wall, at the thoracic inlet (Figure 1). 

It communicated with the tracheal lumen with a narrow opening. 

There was no pneumomediastinum or subcutaneous emphysema. After 2 days, he no longer complained of odynophagia that was related to the ingestion of chicken bone. Our attitude was to continue the follow-up of the tracheocele without surgical intervention. After 3 months of follow-up, the patient was asymptomatic and in the CT scan, the size of the tracheocele was stable.

***Case 2***

**Fig 2:**
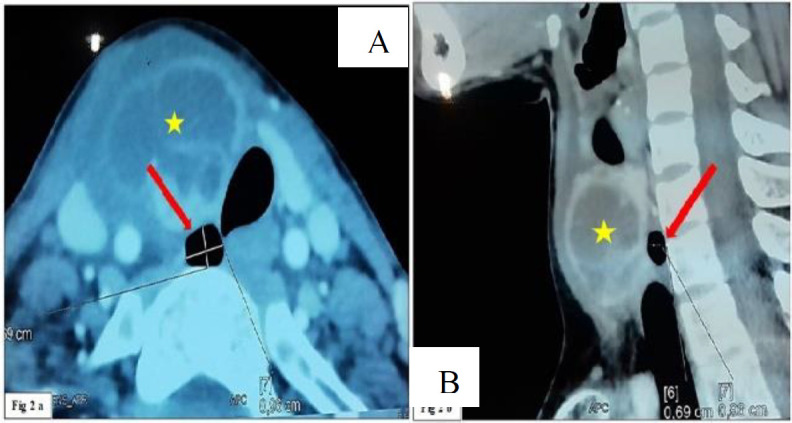
a: Cervical CT (computed tomography) scan in axial section with contrast enhancement shows a ring enhancing cystic lesion located in the right lobe of the thyroid gland ( ) which exerts a mass effect on the trachea, and a 9,6 x 6,9 mm air image ( ) located in the right posterolateral tracheal wall without communication with the lumen of the trachea.. 2b: Cervical CT (computed tomography) scan in sagittal section with contrast enhancement shows a ring enhancing pretracheal cystic lesion ( ) and a 9,6 x 6,9 mm retrotracheal air image ( ).

A 28-year-old woman consulted for anterior neck mass and fever lasting for 2 days. Physical exam showed a painful and inflammatory anterior neck mass moving on swallowing. She had no compressive symptoms. She gave no history of chronic cough or recurrent respiratory infections. The laboratory workup revealed a biological inflammatory syndrome. The CT scan showed a ring enhancing cystic lesion located in the right lobe of the thyroid. Furthermore, it revealed a 9,6 x 6,9 mm air image located in the right posterolateral tracheal wall without obvious communication with the lumen of the trachea (Figure 2).

The barium contrast study did not show an esophageal communication. Ultrasound guided aspiration of the cystic lesion was performed: the fluid was brown and free of bacteria. An infected thyroid hematocele was suspected. A clinical and biological improvement was obtained after 10 days of intravenous antibiotic therapy. Three months later, she underwent a right lobectomy. Intraoperatively, a 1 cm outpouching derived from the right posterolateral wall of the trachea, opposite to the third tracheal ring. The resection of the tracheocele was performed. The postoperative course was uneventful. Histological exam confirmed the diagnosis of tracheocele. Histological exam of the thyroid gland revealed a benign nodule. After 3 years of follow-up, CT scan showed no recurrence of the tracheocele.

## Discussion

A tracheocele is an air-filled outpouching arising from the tracheal wall (1,4). It is a type of paratracheal air cyst (3). It is an uncommon entity; with only few cases have been reported in the literature (2,5). Pharyngeal and laryngeal diverticula are more common (2,6). Tracheocele is usually seen in patients in the sixth decade without consensus about the distribution in terms of gender (2,3). Tracheocele is mainly found in the right para-tracheal region (97.1%) and rarely on the left side (2.9%) (1,3,4). The right preferred location could be related to back support against the esophagus on the left side, which constricts development on this side of the tracheal wall (2,4,7). The tracheocele was located in the right posterolateral tracheal wall in our 2 cases. It can be single or multiple (1.3%) (2,4,5). The most frequent localization is the upper trachea (98%) (2). Tracheoceles can be congenital or acquired. Congenital tracheoceles are extremely rare and formed due to defective differentiation of the endodermal layer of the posterior membrane of the trachea or a defect in the development of the tracheal cartilage (1–3). Therefore, congenital tracheocele is considered as a true diverticulum: a thick wall formed by cartilage, smooth muscle and respiratory epithelium (1,4,6). It mostly affects males and is usually single (1,3,5). The opening of congenital tracheocele is often narrow that can be difficult to see, even by tracheoscopy (1,3,4). It is rarely associated with other congenital malformations such as tracheoesophageal fistula (3,6). Compared to congenital tracheocele , acquired form has a thin wall, lined by a respiratory epithelium only, without smooth muscle or cartilage (1,3,4). Thus, it is a pseudodiverticulum (1). It has an equal sex ratio (5). It is larger and has a wider communication with the tracheal lumen with clear visibility during tracheoscopy (1,2,4). So, acquired form has usually an adequate drainage of secretions and patients are often asymptomatic and infection free (5). Acquired tracheocele is usually found at the level of the thoracic inlet (1,2,7). The main mechanism of acquired variety is the increase in intraluminal tracheal pressure that makes an invagination into a weak tracheal zone (due to repeated respiratory infections) (1,2,4). Thus, the most frequent cause of acquired tracheocele is chronic cough (2). Cough may be an etiological factor or a symptom (7). The other causes are: obstructive lung disease, tracheomalacia, thoracic surgeries and mispositioned endotracheal tube and its cuff pressure (1,3). The tracheocele, in our 2 cases, was probably congenital: no risk factors were noted and the opening of the tracheocele was narrow. Tracheocele may be seen in patients with tracheobronchomegaly or Mounier-Kuhn syndrome and it is frequently multiple (3,7). Tracheocele is usually asymptomatic and mostly found incidentally on radiological investigations or during post-mortem examinations (1,2,4,5). In our cases, the tracheocele was asymptomatic and discovered fortuitously on cervical CT scan done for other reason. When symptoms are present, they are nonspecific and depend on the size and the site of the tracheocele (4,5). 

The common symptoms are chronic cough, stridor or recurrent respiratory infections (2). Dysphagia, odynophagia, dyspnea, dysphonia (by local mass effect or vocal fold paralysis due to impingement on the recurrent laryngeal nerve), hemoptysis, hiccups, burping or neck mass can reveal a tracheocele (2–4,8). The differential diagnosis includes laryngoceles, pharyngoceles, Zenker diverticulum, pulmonary apical hernias, blebs, pneumomediastinum or bullae (2,7).

High-resolution cervicothoracic CT is the best noninvasive technique to detect the tracheocele and evaluate its size and site (3,4,8). On CT scan, tracheocele is described as an air-filled invagination arising from the tracheal wall (4). CT scan shows a communication with the airway in only 12.9 – 16.6% of cases (3). The presence or absence of the cartilage in the wall of the tracheocele and the size of its opening can help to distinguish between congenital and acquired forms (5,6). Chest radiography, bronchography and tracheoscopy are other diagnostic modalities (4). Tracheoscopy is also a useful tool for diagnosing, but tracheoceles with narrow communications or those joined to the trachea by a fibrous tract cannot be visible (3,7,8). Previously, tracheocele was diagnosed by bronchography (4). 

Barium swallow contrast study can help to eliminate a communication with the esophagus, especially in cases of absence of tracheal communication on CT scan. 

Secondary infection is the main complication with the potential risk of a paratracheal abscess (1,3). The air sac may act as a reservoir for secretions, so it becomes a potential source of tracheobronchial infections (5,8). Improper positioning of the endotracheal tube may lead to ventilatory insufficiency or perforation of the tracheocele (3,4). Therefore, intubation should be performed carefully in patients with known tracheocele (4). The treatment options include: observation, fine needle aspiration, surgical resection and endoscopic treatment (fulguration and endoscopic cauterization with a laser or electrocoagulation) (2). 

Conservative treatment includes antibiotics, mucolytic agents, bronchodilators, and physiotherapy (3,4). Due to its rarity, there are no management guidelines of tracheocele. In the absence of symptoms, management is conservative (1–5,7). There is no evidence in the literature for preemptive surgical resection of tracheocele (1). However, patients need to be kept on close follow-up to rule out complications especially para-tracheal abscess (1). Elderly patients are preferably treated conservatively (3–5). Symptomatic and complicated tracheoceles must be treated (2,5). The treatment of choice is surgical resection: effective and safe with excellent result (3,4,6,8). According to Lin et al (6), surgery is indicated only for symptomatic congenital diverticulum, in case of acquired form there is no obvious benefit. The surgical approach varies according to the site and the level of the diverticulum (4,5). The surgeon has to pay attention to avoid damage of the laryngeal recurrent nerve and the esophagus (6). Multiple and wide-based acquired tracheoceles are usually treated conservatively (prevention of the infection) (3).

## Conclusion

Tracheocele is a rare entity. It is frequently located in the right para-tracheal region. The presence or absence of risk factors, CT scan, bronchoscopy and histologic exam may distinguish between congenital and acquired form. Chronic cough is the most frequent cause of acquired tracheocele. It is often asymptomatic and usually discovered incidentally on radiological investigations. CT scan confirms the diagnosis. Tracheoscopy is not necessary for the diagnosis. Paratracheal abscess is the main complication. Asymptomatic patients are managed conservatively. Surgical resection is the treatment of choice for symptomatic patients.
